# Novel Strategies to Reduce Patient No-Show Rates: Single Institutional Study at Jane H. Booker Family Health Center

**DOI:** 10.7759/cureus.105051

**Published:** 2026-03-11

**Authors:** Swapnil V Patel, Jake W Schuler, Jeris Abuhouran, Harshavardhan Sanekommu, Annamarie Cutroneo, Joseph Monica, Renita Luke-Siddon, Harpreet Pall, Vito Buccellato, Kenneth Sable, Joseph Heaton, Natasha Campbell, Mohammad A Hossain

**Affiliations:** 1 Internal Medicine, Jersey Shore University Medical Center, Neptune, USA; 2 Gastroenterology, Jersey Shore University Medical Center, Neptune, USA; 3 Healthcare Administration, Jersey Shore University Medical Center, Neptune, USA; 4 Cardiology, Jersey Shore University Medical Center, Neptune, USA; 5 Medicine, Hackensack Meridian School of Medicine, Nutley, USA

**Keywords:** appointment adherence, care accessibility, healthcare utilization, missed appointments, patient no-shows

## Abstract

Background

Patient no-shows pose a significant healthcare burden, disrupting operational efficiency, reducing access to care, and contributing to poor patient outcomes. The downstream effects of patient no-shows create substantial challenges for healthcare systems, often resulting in wasted resources, decreased productivity, and lost revenue. At the Jane H. Booker Family Health Center in Neptune, New Jersey, patient no-show rates had been steadily rising, prompting the implementation of several targeted interventions.

Methods

Coordinated interventions were implemented, including expanded laboratory hours, a centralized contact center, on-site social work and financial assistance programs, and a structured approach to maintaining consistency across patient-provider relationships. Interventions were introduced in a staggered manner between September 2022 and January 2023 to allow for operational feasibility and progressive implementation. A retrospective analysis of appointment data was conducted, comparing no-show rates before and after implementation of the interventions. These interventions were applied across all specialty and subspecialty services at the center, including Internal Medicine, Pediatrics, Obstetrics, Gynecology, Psychiatry, Endocrinology, Neurology, Pulmonary, Cardiology, Surgery, Nephrology, Urology, Podiatry, Rheumatology, Ophthalmology, and Gastroenterology.

Results

Implementation of these interventions was associated with a measurable improvement in no-show rates. The average no-show rate was 18.6% in 2022, and in 2023, following the interventions, the rate declined to 12.3%, representing an overall 33.8% relative reduction in patient no-show rate (p < 0.001).

Conclusion

A comprehensive, multifaceted intervention strategy was associated with a significant reduction in patient no-show rates. The strategies implemented may serve as a model for other healthcare institutions facing similar challenges. While individual strategies to reduce no-shows have been studied, few examine the combined effect of multiple interventions, highlighting the potential benefits of an integrated approach.

## Introduction

Patient no-shows, which are defined as scheduled appointments that patients fail to attend without prior cancellation, pose a significant challenge for both patients and the healthcare system. A multifaceted intervention was implemented at the Jane H. Booker Family Health Center to address these challenges. Although not a Federally Qualified Health Center (FQHC), the Jane H. Booker Family Health Center serves a patient population similar to that of FQHCs, which function as essential safety-net providers delivering comprehensive primary and preventive care to underserved communities. In New Jersey, 23 FQHCs operate across 138 service sites, collectively serving more than 620,000 patients and providing over two million visits annually. The Jane H. Booker Family Health Center holds a Patient-Centered Medical Home (PCMH) designation and emphasizes team-based, coordinated, and patient-centered care to improve access, continuity, and quality of care. The center offers a broad range of medical and social services, including subspecialty care, to a culturally and socioeconomically diverse patient population. Each year, the center delivers care through more than 30,000 appointments, underscoring both the scale of its reach and the importance of strategies to reduce no-show rates.

A retrospective cohort study of FQHCs across the United States revealed a mean no-show rate of 18.8% over 12 years, with the highest rates occurring in subspecialty clinics [1]. FQHCs face particular challenges that contribute to elevated no-show rates, including limited availability of community resources to support health and socioeconomic stability. Prior studies have demonstrated that social determinants of health, such as transportation insecurity, language barriers, financial hardship, and fragmented care coordination, are strongly associated with missed appointments and reduced healthcare engagement [2,3]. The underutilization of available time slots triggers a cascade of downstream effects, including disrupted workflow, reduced clinical capacity, prolonged wait times for new and established patients, and delayed care. These delays can result in higher emergency room admissions and increased medical expenditures for both patients and the healthcare system. Furthermore, underutilized time slots lead to decreased revenue and operational inefficiencies. Simulation models suggest that 67,000 no-shows could cost the healthcare system approximately $7 million annually [2]. Reducing no-show rates is therefore essential for optimizing workflow and clinical capacity to deliver effective care. Prior health services research has demonstrated that missed outpatient appointments are associated with increased emergency department utilization, higher downstream healthcare costs, and reduced continuity of care, particularly in primary care and safety-net settings [4].

Historically, no-show rates have been addressed using various individual strategies with mixed results. Common interventions include access to interpreter and transportation services, phone call or email reminders, centralized registration areas with self-check-in kiosks, and centralized call centers [3,5]. These approaches have shown variable success when applied in isolation. More recent studies suggest that interventions targeting a single barrier may have limited durability, particularly in patient populations with complex social and medical needs [6,7]. However, a gap remains in the literature regarding a comprehensive, integrated approach that combines multiple strategies simultaneously. By implementing a combination of access, social, financial, and continuity-focused interventions, this study addresses an important gap in understanding how coordinated strategies may influence patient engagement and clinic operations in a large, diverse primary care setting. The primary objective of this study was to evaluate the effect of a staggered, multifaceted intervention on patient no-show rates at the Jane H. Booker Family Health Center. A secondary objective was to assess changes in operational efficiency by comparing appointment outcomes before and after implementation between 2022 and 2023.

## Materials and methods

In response to rising patient no-show rates, a series of coordinated interventions was implemented using a staggered rollout between September 2022 and January 2023. This timeline reflected the administrative and operational realities of program development and rollout, as implementing all five interventions simultaneously would not have been feasible. The interventions included expanded laboratory hours, a centralized contact center for scheduling and patient communication, establishment of on-site social work and financial assistance programs, and promotion of continuity across patient-provider relationships (Table 1). Each strategy was designed to reduce barriers, improve patient engagement, and promote consistent follow-up.

**Table 1:** Multifaceted Interventions Implemented at Jane H. Booker Family Health Center

Strategy	Intervention
Enhanced Laboratory Access	Expanded lab hours from six to 40 hours/week with walk-in availability; patients assigned to one to two consistent providers to improve continuity and care coordination.
Centralized Patient Communication	Centralized contact center to streamline appointment scheduling, patient communication, prescription requests, and follow-up navigation.
Comprehensive Social Support	On-site social workers providing multilingual support, interpretation services, assistance with social services, therapy, and community resource connections.
Financial Assistance and Affordability	On-site financial assistance program; fixed visit fees; free adult and pediatric vaccines; access to free medications through the Pharmacy of Hope for eligible patients.
Continuity and Patient-Provider Engagement	Continuity clinic model ensuring consistent provider teams, including physicians, nurses, social workers, and behavioral health specialists, to help foster strong patient-provider relationships and improve adherence.

This study involved a retrospective analysis of aggregated, de-identified patient appointment data collected as part of routine clinical operations at the Jane H. Booker Family Health Center. No direct patient contact occurred, and no individually identifiable information was accessed or analyzed. All data were handled in accordance with institutional policies for patient privacy and confidentiality and were used solely for the purposes of healthcare quality improvement and operational evaluation. 

Inclusion criteria consisted of all scheduled outpatient appointments across participating clinics during calendar years 2022 and 2023. Exclusion criteria included appointments canceled in advance by patients, appointments rescheduled prior to the visit date, and visits scheduled outside the study period. A no-show was defined as any scheduled appointment that was neither completed nor canceled in advance by the patient. The sample size was determined by the inclusion of all eligible appointments during the study period. Interventions were selected based on review of clinic operational data, patterns of missed appointments, and input from frontline clinical and administrative staff, which identified access barriers, communication challenges, financial hardship, and lack of continuity of care as the most common contributors to patient no-shows.

Expansion of laboratory services

Laboratory operating hours were expanded to Monday through Friday, from 7:30 AM to 3:30 PM, compared to the previous schedule of three two-hour sessions per week. Restrictions based on payer source and patient age were removed to increase access for all patients. The laboratory is accredited under the Clinical Laboratory Improvement Amendments (CLIA), allowing comprehensive testing for COVID-19, influenza, respiratory syncytial virus (RSV), and other infectious diseases. Strategic partnerships with LabCorp and Quest Diagnostics provided a reliable supply of testing materials at minimal or no cost, supporting financial sustainability. For uninsured patients, each lab visit was offered at a fixed rate of $34, with no additional balance billed, improving affordability and reducing barriers to care. Laboratory technicians received electronic medical record (EMR) training to register walk-in patients efficiently. Oversight by a dedicated laboratory coordinator ensured consistent workflow, rapid test processing, and quality control, allowing the lab to accommodate increased patient volume without compromising accuracy or efficiency.

Centralized contact center

A centralized contact center was implemented to streamline patient communication and reduce confusion associated with navigating multiple phone lines and extensions. The center now serves as a single point of contact for all patient needs, including appointment scheduling, prescription refills, laboratory inquiries, referral follow-up, and after-hours provider contact. Staffed by trained medical assistants, the center provides multilingual support in English, Spanish, and French-Creole, with certified interpreters available to overcome language barriers. The center also manages high call volume and addresses patient health literacy by providing clear explanations of care instructions and test results. Workflow efficiency was further enhanced through structured training programs and standardized protocols, ensuring timely responses to patient inquiries and improved overall satisfaction.

On-site social work services

The on-site social work team expanded from three to seven professionals, with revised roles to provide a broader range of services. The team includes two case managers, one mental health specialist, one behavioral health specialist, and three licensed social workers (one LSW and two LCSWs), with language-certified staff addressing Spanish and French-Creole needs. Services include assistance with food programs, WIC, SNAP, utility support, therapy sessions, and referrals to community-based resources. Case managers guide patients through paperwork and enrollment in supportive programs, while two social workers conduct weekly outreach to local community organizations. These efforts address social determinants of health, such as food insecurity, housing, and financial stress, which are critical contributors to missed appointments.

Financial assistance program

A structured financial assistance program was introduced in collaboration with the social work team to enhance affordability and support patients in navigating healthcare costs. Upon registration, patients are referred to the financial assistance office for individualized guidance, including education on billing, charity care, and payment options. Standardized visit fees ($80 for general visits and $95 for subspecialty visits) with no balance billing reduce unexpected financial burdens. Free vaccines for adults and children and access to the Pharmacy of Hope for eligible uninsured patients further mitigate economic barriers, supporting continuity of care and adherence.

Consistency across patient-provider relationships

The continuity clinic model was restructured to ensure patients consistently see the same provider or a small provider team, fostering trust, collaboration, and stronger therapeutic relationships. Each team typically includes primary care physicians, nurse practitioners, medical assistants, registered nurses, social workers, and behavioral health specialists. By maintaining continuity, teams can better monitor patient progress, anticipate care needs, and provide personalized interventions. This collaborative, team-based approach integrates medical care, behavioral health, and social services, promoting adherence, improving patient satisfaction, and addressing barriers that contribute to missed appointments.

Data collection and analysis

Data were collected across all clinical specialties, including Internal Medicine, Pediatrics, Obstetrics and Gynecology, Psychiatry, Endocrinology, Neurology, Pulmonary, Cardiology, Surgery, Nephrology, Urology, Podiatry, Rheumatology, Ophthalmology, and Gastroenterology. No-show rates were calculated as the proportion of scheduled visits that were completed, before and after implementation of the multifaceted interventions. Appointment outcomes were categorized as completed visits or no-shows and treated as categorical variables for analysis. Aggregated data enabled assessment of overall clinic performance and identification of areas where operational and patient-focused interventions had the greatest impact, facilitating continuous quality improvement. Comparisons of appointment outcomes between 2022 and 2023 were performed using a chi-square test of independence to compare proportions of categorical outcomes between the two time periods. Statistical significance was defined as a two-sided p-value < 0.05. Appointment outcomes were analyzed in aggregated form at the clinic level rather than as paired patient-level data. Statistical analyses were performed using standard spreadsheet-based calculations.

## Results

Following the implementation of the interventions at the Jane H. Booker Family Health Center, aggregate data were analyzed to assess the impact on patient no-show rates and operational efficiency. The interventions were introduced in a staggered manner between September 2022 and January 2023, allowing gradual integration of each initiative and contributing to a steady decline in no-show rates beginning in late 2022. In 2022, a total of 30,307 patient appointments were scheduled, with 24,186 completed visits, corresponding to an average no-show rate of 18.6%. In 2023, 31,887 appointments were scheduled, with 27,870 completed, resulting in an average no-show rate of 12.3%. These results represent a 33.8% relative reduction in the average patient no-show rate between 2022 and 2023, demonstrating a substantial improvement in patient engagement and appointment adherence. This sustained improvement throughout 2023 suggests the cumulative impact of sequentially implemented interventions.

A chi-square test of independence comparing appointment outcomes (completed visits versus no-shows) between 2022 and 2023 demonstrated a statistically significant reduction in no-show rates (χ² = 536.18, p < 0.001). The large sample size and highly significant p-value suggest that this reduction was both statistically and clinically meaningful.

**Figure 1:**
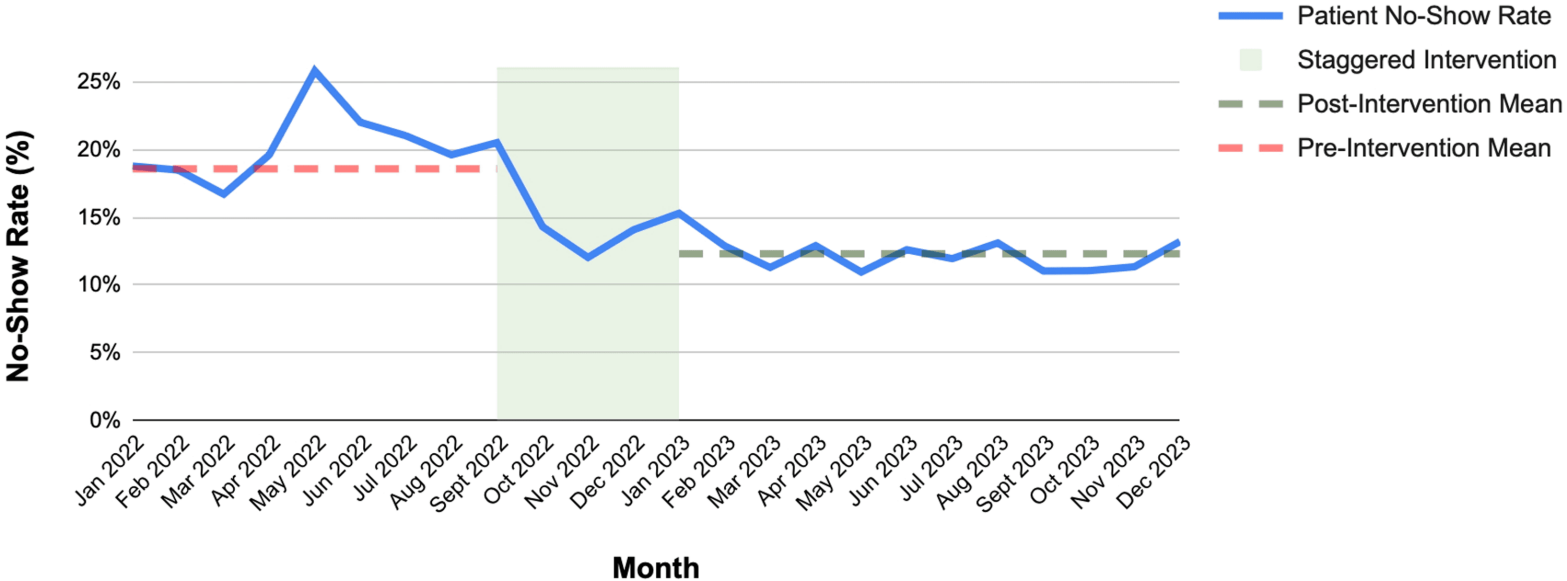
Monthly Patient No-Show Rates Before and After Interventions Monthly patient no-show rates for 2022 and 2023 are presented, showing a consistent decrease in missed appointments across nearly all months following the staggered implementation of interventions between September 2022 and January 2023. The figure highlights the effectiveness of the sequential, multifaceted strategies in maintaining lower no-show rates throughout 2023. Image made using Microsoft Excel (Microsoft® Corp., Redmond, WA) and Google Sheets (Google, Inc., Mountain View, CA).

**Figure 2:**
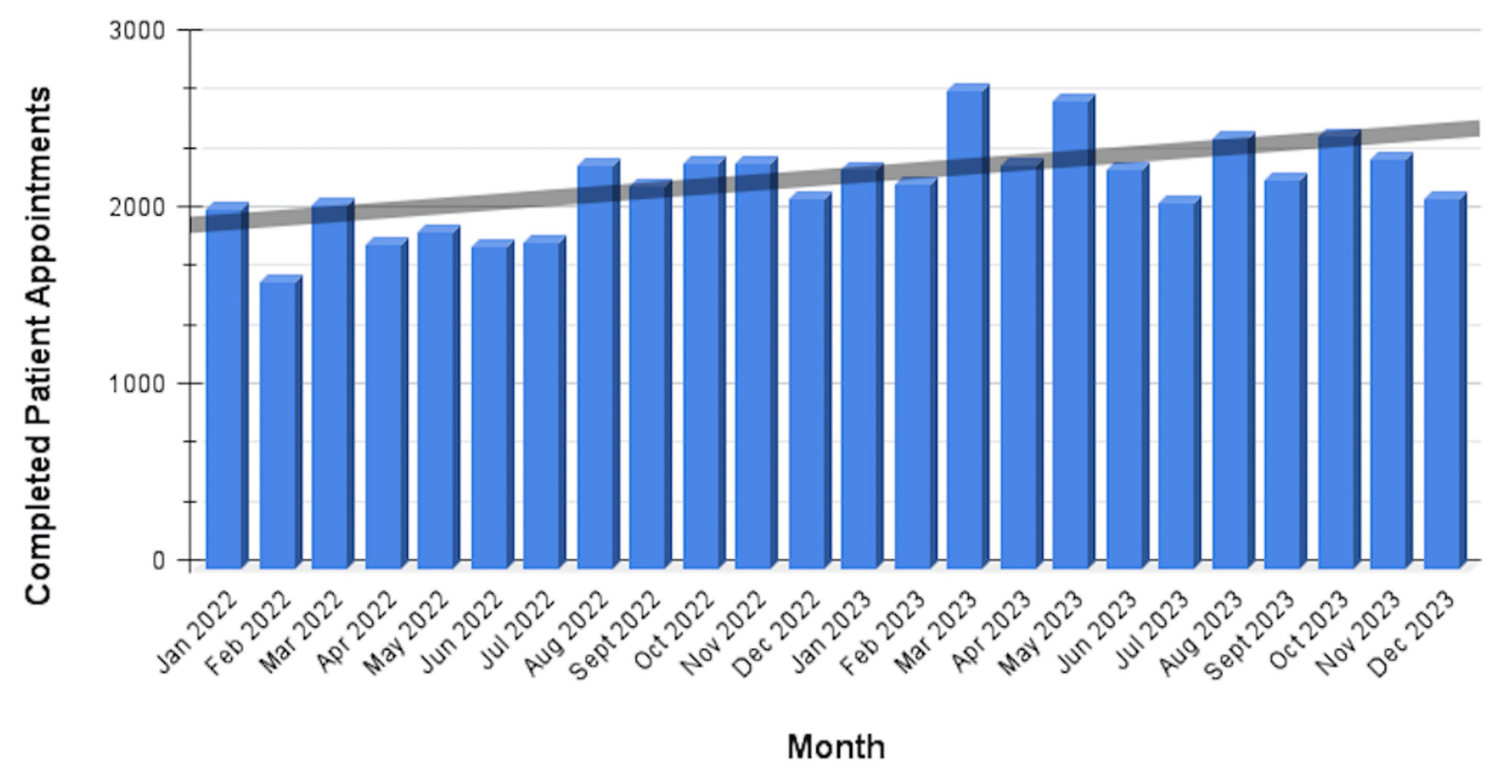
Total Completed Patient Appointments, 2022-2023 The total number of completed patient appointments for 2022 (24,186) and 2023 (27,870) is depicted by month. The increase in completed visits over time after a multifaceted intervention reflects enhanced operational efficiency, improved accessibility of care, and successful reduction of barriers to attendance. Image made using Microsoft Excel (Microsoft® Corp., Redmond, WA) and Google Sheets (Google, Inc., Mountain View, CA).

**Figure 3:**
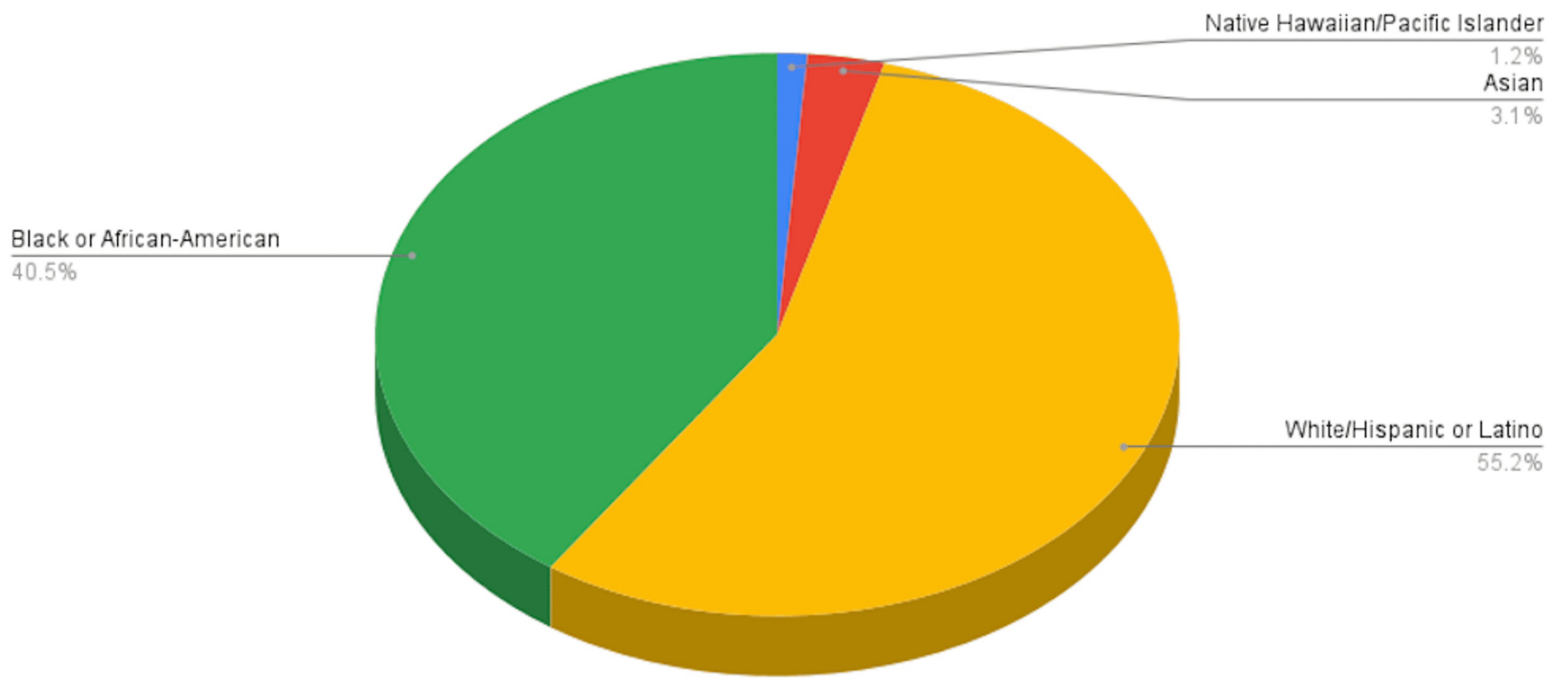
Demographic Distribution of Patients Across Specialties The demographic distribution of patients across all specialties and subspecialties at the center is displayed. The data demonstrate the diversity of the patient population in terms of age, gender, and cultural background, emphasizing the importance of tailored, patient-centered strategies to reduce no-show rates in a heterogeneous community setting. Image made using Microsoft Excel (Microsoft® Corp., Redmond, WA) and Google Sheets (Google, Inc., Mountain View, CA).

Figures 1-3 illustrate trends over time, changes in completed appointments, and the demographic profile of the patient population served. 

## Discussion

This single-center study implemented a series of coordinated interventions, resulting in an overall 33.8% relative reduction in average no-show rates at the Jane H. Booker Family Health Center between 2022 and 2023. Although not designated as an FQHC, the health center serves a patient population and fulfills a mission closely aligned with FQHCs, providing comprehensive, team-based care to improve accessibility, continuity, and coordination. The center also maintains PCMH recognition, emphasizing coordinated and patient-centered service delivery. Within this framework, interventions such as expansion of laboratory hours, establishment of a centralized contact center, on-site social work and financial assistance programs, and consistency in patient-provider relationships were associated with meaningful improvements in appointment adherence. While some of these strategies have been studied individually, few publications examine their combined implementation, highlighting the synergistic benefit of a multifaceted approach. Importantly, these interventions were implemented in a staggered fashion, reflecting real-world administrative constraints and allowing progressive integration of each component into routine clinical operations.

Although these results demonstrate a substantial and statistically significant improvement in appointment adherence, the study design limits the ability to draw definitive causal conclusions. While the reductions in no-show rates are temporally associated with implementation of the interventions, the pre-post design does not permit isolation of their individual effects or confirmation of direct causality. The findings should therefore be interpreted as demonstrating an association between the multifaceted intervention strategy and improved appointment adherence rather than proof of effectiveness of any single component. Nevertheless, the magnitude and consistency of the reduction across the post-intervention period support the conclusion that a coordinated, system-level approach can meaningfully improve operational performance and patient engagement in outpatient settings.

A review of existing literature demonstrates that no-show rates range from 12% to 80% across healthcare settings [2,3]. In 2022, the no-show rate at the Jane H. Booker Family Health Center reached as high as 26%, fluctuating between 16% and 25% for most of the year. Following implementation of the interventions, the average no-show rate decreased substantially and remained between 10% and 14% throughout 2023. Similar interventions have shown positive effects in other studies. For example, targeted patient navigation and interpreter services reduced no-show rates among Spanish-speaking families from 28% to 17% [5,6]. Centralized reminder systems using phone, text, or email have been associated with reductions in missed appointments ranging from 20% to 25% [7-10]. The magnitude of reduction observed in the present study is comparable to, and in some cases greater than, improvements reported in these prior studies evaluating reminder systems, patient navigation programs, and bundled access interventions, supporting the clinical relevance of the observed effect. In a recent randomized controlled quality improvement initiative, predictive model-driven live reminder calls reduced no-show rates from 36% to 33% and narrowed racial and ethnic disparities in appointment attendance [7]. On-site social work and financial assistance programs have also demonstrated improved patient adherence, particularly among populations facing socioeconomic barriers [5,6]. Systematic reviews suggest that bundled or multi-component interventions addressing both operational and social determinants of health are more likely to produce sustained reductions in no-show rates compared with single-strategy approaches [4]. Collectively, these findings indicate that coordinated, multifaceted strategies can significantly enhance patient engagement and reduce missed appointments across diverse healthcare settings.

From a healthcare system perspective, patient no-shows represent substantial financial losses. Hospitals with high no-show rates may experience operational inefficiencies, underutilized resources, and reduced revenue, particularly in clinics equipped with specialized testing or advanced technologies. One study reported an average of 62 missed appointments per day at a community hospital, translating into an estimated annual loss of $3 million [1]. Another study estimated that missed appointments across the Veterans Health Administration could cost over $564 million annually [1]. Beyond the financial implications, missed appointments delay diagnosis and treatment, prolong wait times, and reduce access for other patients awaiting care. These disruptions may lead to increased emergency department utilization for non-urgent conditions and encourage overbooking practices that strain clinical workflows [9,10]. By improving appointment adherence, the interventions described may help mitigate both economic inefficiencies and downstream clinical consequences associated with delayed care. These findings are consistent with broader health services literature demonstrating that outpatient no-shows contribute to avoidable utilization, inefficiencies, and increased costs across ambulatory care delivery systems [2,4].

This study has several limitations. Patient demographics were included but not fully stratified. Understanding differences in socioeconomic status, race, age, gender, and ethnicity is crucial, as these factors may influence no-show rates. The absence of a formal root cause analysis prior to intervention selection, the potential influence of unmeasured co-interventions, and the use of a pre-post study design without a control group may limit causal inference and may require cautious interpretation of the observed reductions in no-show rates. Furthermore, because demographic and socioeconomic variables were not fully stratified at the patient level, differential impacts across subpopulations could not be assessed. The population at the Jane H. Booker Family Health Center may differ from other healthcare settings, limiting generalizability. Additionally, the interventions were implemented in a staggered manner between September 2022 and January 2023, which, while reflecting a realistic and pragmatic rollout process, makes it difficult to isolate the effect of each intervention individually. As a single-center study conducted over one year, the findings may not capture longer-term effects or the sustainability of outcomes. Future studies using controlled designs could help delineate the relative contribution of individual components within multifaceted interventions.

Despite these limitations, the study demonstrates notable strengths. The interventions were clearly defined, integrated across multiple care domains, and implemented within a PCMH framework that emphasizes continuity, accessibility, and comprehensive care coordination. Monthly tracking of no-show rates enabled precise assessment of intervention impact over time. The observed reductions in no-show rates have the potential to mitigate financial losses, improve operational efficiency, and enhance both patient satisfaction and clinical outcomes. Furthermore, this study highlights how simultaneous, complementary strategies, rather than isolated efforts, can produce meaningful and sustainable improvements in appointment adherence and care delivery. These findings add to a growing body of evidence showing that patient-focused strategies at the system level can improve healthcare accessibility and utilization.

## Conclusions

The implementation of a multifaceted, patient-centered approach at the Jane H. Booker Family Health Center was associated with a substantial improvement in average patient no-show rates, with an overall relative reduction of 33.8% between 2022 and 2023. The combination of interventions, including expanded laboratory hours, a centralized contact center, on-site social work and financial assistance programs, and consistent patient-provider relationships, helped address multiple barriers to care and improve appointment adherence. These findings suggest that coordinated, system-level strategies may improve operational efficiency and patient engagement in outpatient settings. Further research is needed to evaluate the long-term sustainability of these interventions and their applicability across other healthcare systems.

## References

[1] Kheirkhah P, Feng Q, Travis LM, Tavakoli-Tabasi S, Sharafkhaneh A (2016). Prevalence, predictors and economic consequences of no-shows. BMC Health Serv Res.

[2] Marbouh D, Khaleel I, Al Shanqiti K (2020). Evaluating the impact of patient no-shows on service quality. Risk Manag Healthc Policy.

[3] Naimi B, Agarwal P, Ma H, Levi JR (2023). Association between no-show rates and interpreter use in a pediatric otolaryngology clinic. Int J Pediatr Otorhinolaryngol.

[4] Ziemba JB, Arenberg S, Reustle H, Allaf ME, Haldeman D (2019). Consumers' association of hospital reputation with healthcare quality. J Healthc Qual.

[5] Flower KB, Wurzelmann S, Rojas C (2020). Improving satisfaction and appointment attendance through navigation for Spanish-speaking families. J Health Care Poor Underserved.

[6] Shour A, Onitilo AA (2023). Distance matters: investigating no-shows in a large rural provider network. Clin Med Res.

[7] Tarabichi Y, Higginbotham J, Riley N, Kaelber DC, Watts B (2023). Reducing disparities in no show rates using predictive model-driven live appointment reminders for at-risk patients: a randomized controlled quality improvement initiative. J Gen Intern Med.

[8] Teo AR, Niederhausen M, Handley R (2023). Using nudges to reduce missed appointments in primary care and mental health: a pragmatic trial. J Gen Intern Med.

[9] Aljuaid MA, Li J, Lin C (2023). Does the combination of phone, email and text-based reminders improve no-show rates for patients in breast imaging?. Curr Probl Diagn Radiol.

[10] Davies ML, Goffman RM, May JH, Monte RJ, Rodriguez KL, Tjader YC, Vargas DL (2016). Large-scale no-show patterns and distributions for clinic operational research. Healthcare (Basel).

